# The Influence of Polycation and Counter-Anion Nature on the Properties of Poly(ionic liquid)-Based Membranes for CO_2_ Separation

**DOI:** 10.3390/membranes13060539

**Published:** 2023-05-23

**Authors:** Ksenia V. Otvagina, Alexey A. Maslov, Diana G. Fukina, Anton N. Petukhov, Yulia B. Malysheva, Andrey V. Vorotyntsev, Tatyana S. Sazanova, Artem A. Atlaskin, Alexander A. Kapinos, Alexandra V. Barysheva, Sergey S. Suvorov, Ivan D. Zanozin, Egor S. Dokin, Ilya V. Vorotyntsev, Olga V. Kazarina

**Affiliations:** 1 Chemical Engineering Laboratory, Research Institute for Chemistry, N.I. Lobachevsky State University of Nizhny Novgorod, 23 Gagarin Avenue, 603950 Nizhny Novgorod, Russia; k.v.otvagina@gmail.com (K.V.O.); antopetukhov@gmail.com (A.N.P.); an.vorotyntsev@gmail.com (A.V.V.); kapinos98@gmail.com (A.A.K.); aleksandra.barysheva@gmail.com (A.V.B.); e-dokin@yandex.ru (E.S.D.); 2 Research Institute for Chemistry, N.I. Lobachevsky State University of Nizhny Novgorod, 23 Gagarin Avenue, 603950 Nizhny Novgorod, Russia; dianafuk@yandex.ru; 3 Laboratory of SMART Polymeric Materials and Technologies, Mendeleev University of Chemical Technology, 9 Miusskaya Square, 125047 Moscow, Russiaatlaskin@gmail.com (A.A.A.); ilyavorotyntsevv@gmail.com (I.V.V.); 4 Organic Chemistry Department, N.I. Lobachevsky State University of Nizhny Novgorod, 23 Gagarin Avenue, 603950 Nizhny Novgorod, Russia; 5 Laboratory of Membrane and Catalytic Processes, Nizhny Novgorod State Technical University n.a. R.E. Alekseev, 24 Minin Street, 603950 Nizhny Novgorod, Russia; 6 Laboratory of Ionic Materials, Mendeleev University of Chemical Technology, 9 Miusskaya Square, 125047 Moscow, Russia

**Keywords:** polymeric ionic liquids, carbon dioxide, gas separation, composite membranes

## Abstract

The current investigation is focused on the development of composite membranes based on polymeric ionic liquids (PILs) containing imidazolium and pyridinium polycations with various counterions, including hexafluorophosphate, tetrafluoroborate, and bis(trifluoromethylsulfonyl)imide. A combination of spectroscopic methods was used to identify the synthesized PILs and characterize their interaction with carbon dioxide. The density and surface free energy of polymers were performed by wettability measurements, and the results are in good agreement with the permeability and selectivity obtained within the gas transport tests. It was shown that the membranes with a selective layer based on PILs exhibit relatively high permeability with CO_2_ and high ideal selectivity CO_2_/CH_4_ and CO_2_/N_2_. Additionally, it was found that the type of an anion significantly affects the performance of the obtained membranes, with the most pronounced effect from bis-triflimide-based polymers, showing the highest permeability coefficient. These results provide valuable insights into the design and optimization of PIL-based membranes for natural and flue gas treatment.

## 1. Introduction

Modern society is moving towards the transition to the sixth technological paradigm, sustainable technologies, and ESG (Environmental, Social and Corporate Governance) [1]. In this regard, chemical engineering is actively searching for economically beneficial and environmentally friendly approaches to meet the current needs of holistic chemical and petroleum production. Membrane technologies are in a large part considered the main alternative to traditional methods of purification and separation of substances due to their high energy efficiency. This is because they enable continuous processes without the need to replace or regenerate the chemical sorbent [2]. Moreover, membrane processes’ hardware is compact, and easy to operate, manage, and scale when compared to sorption separation of substances. These advantages have caused the rapid development of membrane technologies since the 1960s [3]. Since then, the variety of membranes and membrane systems has grown more than a hundredfold, and the annual production of membrane materials exceeds several tens of millions of square meters. Industrial applications of membrane processes fall into six main groups: reverse osmosis, ultrafiltration, microfiltration, electrodialysis, gas separation, and pervaporation [4]. Baromembrane processes, which include the first three groups, as well as the use of electrodialysis for the desalination of brackish groundwater, are the most commercially developed areas of membrane technology [5]. However, the commercial application of diffusion processes—gas separation and pervaporation—is also growing, with nearly twenty companies worldwide implementing membrane gas separation and pervaporation systems. The potential of membrane applications for obtaining high-purity gases for microelectronics, the production of liquid nitrogen, hydrogen purification for ammonia production, the drying of organic solvents, and especially acid gas and other impurities separation from natural gas has led to a scientific trend in the study of diffusion processes [6].

Currently, the leading industrial application of diffusion processes is natural gas pre-pipeline treatment [7]. Although the composition of natural gas varies depending on the certain source, the main impurities, such as water, carbon dioxide, nitrogen, and hydrogen sulfide, are always present in the stream. Membrane gas separation techniques are mostly focused on removing acid gases from light alkanes, particularly methane. Carbon dioxide is the main target for membrane separation due to its significant presence in the vast majority of natural gas sources. The presence of CO_2_ can cause undesirable issues, such as corrosion and a reduction in the energy impact of the fuel [8]. Besides the natural gas treatment, membrane technology is also applicable to CO_2_ separation from flue gas and biogas.

Polymeric materials currently dominate the market in membrane-based gas separations [6], driving further development of polymer membrane science. Several types of polymer materials have been extensively studied for CO_2_ separation and capture, including polymeric ionic liquids, rubbery polymers with a high content of ether oxygen, perfluoropolymers, and polymers of intrinsic microporosity, among others [9]. In this study, we focus on polymeric ionic liquids as a promising type of polymer matrix for membranes, providing high CO_2_/CH_4_ or CO_2_/N_2_ selectivity.

Ionic liquids (ILs) have been attracting much attention in the field of membrane gas separation [10] since the first report of CO_2_ capture by this class of salts [11]. Liquid at ambient conditions ILs, also known as room temperature ionic liquids (RTILs) [12], have been widely studied as liquid phase absorbent of CO_2_ in supported liquid membranes (SILMs) [13,14,15,16,17,18], mixed matrix membranes (MMMs) [19] and in advanced materials based on inorganic oxides for CO_2_ capture [20,21]. SILMs are obtained by impregnating RTILs into porous polymer supports, and MMMs are synthesized by mixing RTILs with polymer solution before membrane casting. While ILs-based membranes have shown great potential for CO_2_/N_2_ and CO_2_/CH_4_ separations [13,14,15], they cannot withstand high transmembrane pressure, as RTILs tend to leak out from a porous support [22]. To solve this problem, their polymerized analogues—polymeric ionic liquids (PILs)—have been implemented instead of low molecular weight substances, allowing the combination of macromolecular features with the unique properties of ILs [23,24,25].

PILs are formally composed of “IL’s moieties” covalently linked into a polymer backbone. Two main strategies are used for PILs synthesis: (1) the polymerization of the corresponding monomeric IL [26,27,28,29] or (2) the modification a polymer precursor [30,31,32,33,34]. Although the first route allows precise control of polymer composition and structure, the polymerization process is complicated by the redistribution of electron density caused by ionic groups, which limits the achievement of high degrees of conversion during polymerization and obtaining high molecular weight polymers [28]. The second approach allows the synthesis of high molecular weight PILs, but the composition of the polymer may not be entirely uniform due to the less than 100% degree of modification caused by steric hindrance of the polymer chains [34]. Since the pioneering work was published by Nobel’s group [35], PILs have been extensively studied as a membrane material in a gas separation field [10,36,37,38,39]. Although some PILs in combination with ILs exhibit extraordinary selectivity for CO_2_/CH_4_ and CO_2_/N_2_ gas pairs and overcome the Robeson upper bound [40,41], they also have some disadvantages as a membrane material. Most PILs proposed as membrane matrix for CO_2_ separation form brittle films incapable of withstanding the pressure drop of the gas flow. Another problem is the drastic decrease of CO_2_ permeability in the direct ratio to polymer molecular weight [42]. However, a wide range of possibilities for polymer design through a reasonable choice of monomeric units, as well as polymer structure, may significantly improve a polymer’s performance as a membrane material for CO_2_ separation.

In the case of PILs, both the type of ions and the nature of the polymer backbone play a significant role in membrane separation performance [28,43,44,45]. Recently, polymerizable ILs based on vinylbenzyl chloride (VBCl) modified with ammonium moieties have been proposed and investigated as CO_2_ adsorbents and gas separation membrane matrices [27,44,46,47,48,49,50]. Noble’s group has shown that styrene-based PILs are more CO2-selective than vinyl-based PILs [48]. Polystyrene is indeed a good choice for a PIL backbone due to its exceptional mechanical properties and relatively high T_g_. Incorporating ionic substitutes undoubtedly influences the overall properties of PILs, however, styrene and VBCl are cheap, available, and easy-to-modify starting materials. In Table 1, we have summarized the gas separation properties of membranes synthesized from VBCl homopolymer derivatives that have been previously reported in the literature.

Although some advantages of applying styrene-based PILs in membrane design have been demonstrated, there remains a need for a deeper understanding of how ionic composition affects CO_2_ separation performance for this class of polyelectrolytes. This work focuses on the synthesis and characterization of PILs with polystyrene as the polymer backbone, utilizing either methylimidazolium or pyridinium cationic substitutes and a range of counter-anions: chloride [Cl], tetrafluoroborate [BF_4_], hexafluorophosphate [PF_6_], and bis(trifluoromethylsulfonyl)imide [Tf_2_N]. Composite gas separation membranes based on the synthesized PILs were prepared and tested for individual gases (CO_2_, CH_4_, and N_2_) permeation to investigate the influence of polycation functionality and anion type on the CO_2_ separation performance of styrene-based PILs.

## 2. Materials and Methods

### 2.1. Materials

The following reagents were used for PILs synthesis and membrane preparation: 4-vinylbenzyl chloride (VBCl, 90%, Sigma Aldrich, Darmstadt, Germany) was used after purification by vacuum distillation, azobisisobutyronitrile (98%, Chemical line) was used after purification by recrystallization from a dry ethanol solution. Reagents: pyridine (99%), 1-methylimidazole (99%); salts: sodium tetrafluoroborate (NaBF_4_, 98%), potassium hexafluorophosphate (KPF_6_, >99%), lithium bis(trifluoromethylsulfonyl)imide (LiTf_2_N, >99%) were purchased from Sigma-Aldrich; organic solvents: toluene (≥99.5%), chloroform (≥99.5%), isopropyl alcohol (≥98%), diethyl ether (≥99%), dimethylsulfoxide (DMSO, ≥99%) were procured from Chimreaktiv and used without additional purification. Water was purified by double distillation. A commercial microfiltration fluoroplastic membrane (MFFK-hydrophilic, pore size 0.15 µm by Vladipor, Vladimir, Russian Federation) was used as a support for composite membrane preparation.

### 2.2. Poly(vinylbenzyl chloride) (pVBCl) Synthesis

pVBCl was synthesized via free radical polymerization in mass according to a well-established technique. The procedure was as follows: initiator AIBN (0.05 g) was dissolved in VBCl (20 g) and placed into an ampule. The ampule was frozen in liquid nitrogen, exposed to vacuum and then defrosted. The freezing-vacuuming treatment was repeated three times. Then, the ampule was sealed under vacuum. Polymerization was carried out for 12 h at 70 °C in an oil bath. The product of polymerization was purified by dissolution in chloroform followed by precipitation with isopropanol. Dissolving-precipitation treatment was repeated three times. The resulting pVBCl was dried under vacuum at 60 °C to constant weight. The yield of the product was 91%.

### 2.3. Poly(vinylbenzylpyridinium chloride) (pVBPyCl) and Poly(vinylbenzylmethylimidazolium chloride) (pVBmimCl) Synthesis

The obtained pVBCl was used as a precursor for pVBPyCl, pVBmimCl synthesis by Menshutkin reaction. For pVBPyCl synthesis, pVBCl (4 g) was dissolved in pyridine (10 mL). For pVBmimCl synthesis, pVBCl (4 g) was dissolved in toluene (10 mL) and a 10 mol% excess of 1-methylimidazole was added under constant stirring. The reaction was carried out for 12 h at 60 °C with reflux in an oil bath. The products were purified by decantation followed by threefold washing of the precipitate in diethyl ether. The resulting products were dried under vacuum at 50 °C to constant weight. The yield of pVBPyCl and pVBmimCl was 96% and 92%, respectively. Conductometric titration was performed to determine the functionalization degree (FD) as it is described in Section 2.5.

### 2.4. Anion Exchange Reaction

**Scheme 1:**
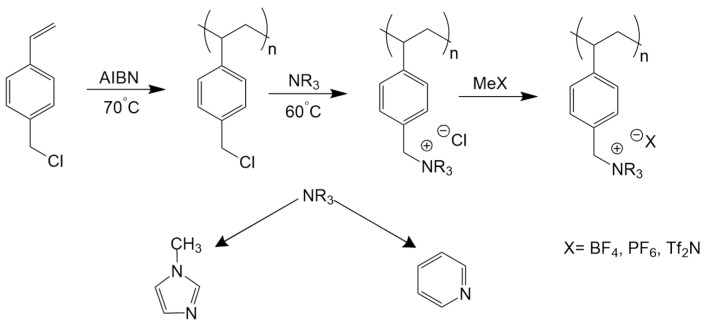
General synthetic route for PIL synthesis.

A synthetic route for PIL synthesis is demonstrated in Scheme 1. A series of PILs (*pVBPyBF*_4_, *pVBPyPF*_6_, *pVBPyTf*_2_*N*, *pVBmimBF*_4_, *pVBmimPF*_6_, *pVBmimTf*_2_*N*) with various counterions was synthesized from pVBPyCl and pVBmimCl by ion exchange reactions with one of the following salts: NaBF_4_, KPF_6_, or LiTf_2_N. The procedure for all PILs was as follows: a weighted amount of polyelectrolyte with Cl-anion was dissolved in water in a flask, and a 10 mol% excess of salt solution in water was gradually added under constant stirring. The reaction products were insoluble in water and precipitated immediately. Due to the polymeric nature of cations, the reaction was carried out for 24 h at 25 °C with stirring to ensure the maximum possible exchange. The product was isolated by filtering using a Buchner filter funnel with fine frit (G3) and purified by washing three times in distilled water. Following, drying under vacuum at 40 °C to constant weight was performed. Conductometric titration was performed to determine the anion exchange degree (ExD) as it is described in Section 2.5.

### 2.5. Conductometric Titration 

Conductometric titration was used to confirm and quantify pVBCl functionalization as a functionalization degree (FD), the ratio of the experimentally determined amount of substituent groups to the calculated amount expressed in percentage, and the anion exchange degree (ExD), the ratio of the experimentally determined amount of exchanged anions to the calculated one expressed in percentage. The measurements were performed using a Aquasearcher benchtop meter (OHAUS, NJ, USA) equipped with STCON3 conductivity electrodes (OHAUS, NJ, USA). FD was determined by pVBPyCl or pVBmimCl solution in water (0.0001 M, 100 mL) titration with AgNO_3_ 0.0001 M solution. The measurements were carried out in intervals of 1 mL and 0.2 mL near the equivalence point. Titration was performed 3 times; the average value was calculated. To evaluate ExD, solutions of Cl-containing inorganic salts, formed as a result of an ion exchange reaction, were titrated with AgNO_3_ 0.0001 M solution in water. The measurements were carried out in intervals of 1 mL and 0.2 mL near the equivalence point. Titration was performed 3 times; the average value was calculated.

### 2.6. Nuclear Magnetic Resonance Spectroscopy (NMR) 

The ^1^H NMR spectra were recorded in DMSO-d_6_ solution (99.9% atom D, Sigma-Aldrich) on a DD2 400NB NMR spectrometer (Agilent, CA, USA) at 400 MHz. The residual solvent peak was used as an internal standard. The chemical shifts (δ) are reported in parts per million (ppm); J values are given in hertz (Hz).

### 2.7. Gel Permeation Chromatography (GPC)

Molecular weight (number-average molecular weight (M_n_) and weight-average molecular weight (M_w_)), as well as polydispersity (M_w_/M_n_) of pVBCl were determined by means of GPC on Prominence LC-20VP (Shimadzu) equipped with a RI-detector. The analyses were performed at a flow rate of 0.7 mL·min^− 1^ and at 40 °C using THF (≥99.9%, HPLC quality, Sigma-Aldrich) as an eluent. The GPC system was calibrated using the narrow-dispersed polystyrene (PS) ranging (Fluka).

### 2.8. Attenuated Total Reflectance Fourier Transforms Infrared Spectroscopy (ATR-FTIR)

IR spectra of the samples were recorded using an FTIR spectrophotometer IRTracer-100 (Shimadzu, Kyoto, Japan) equipped with modified HATR accessory with a ZnSe crystal plate (PIKE, NC, USA) in transmittance mode at ambient temperature. The mirror system of the accessory was constantly exposed to a N_2_ flow in order to exclude atmospheric CO_2_ from registered spectra. As it was previously reported [52], HATR accessory was modified with inlet and outlet gas valves in the top cover enabling in situ experiments of gas (CO_2_) sorption by the sample in the cell. The sample of polymer for FTIR analysis was prepared by polymer solution (1 mass% in DMSO) casting on a ZnSe crystal plate followed by solvent evaporation under vacuum at 40 °C. Three types of spectra were recorded for all polymer samples: (1) pure polymer, (2) in situ CO_2_ sorption, (3) polymer after CO_2_ desorption. A minimum of 30 scans was signal-averaged with a resolution of 4 cm^−1^ within the 4000–500 cm^−1^ range.

### 2.9. Density of Polymers

The density of the polymer films obtained by polymer solution (2 mass% in DMSO) casting on inert glass support was measured by implementing a flotation method [53,54]. To conduct an experiment, a graduated cylinder (50 cm^3^) with a ground-in glass stopper was filled to half with a mixture of two miscible liquids with different densities at a 1:1 volume ratio. Particular liquids were chosen based on the following requirements: (1) liquids should be chemically inert to the polymer material, (2) liquids should not cause swelling or solvation of the polymer, (3) the mixture should cover the supposed density range for the polymer. Mixtures of liquid used for different PILs are listed in Table S1. A sample of the polymer film was immersed in the mixture of liquids and brought to an equilibrium middle position, according to the graduation, by adding one of the liquids dropwise. Then, the density of the obtained mixture of liquids was measured using a pycnometer (10 cm^3^). Density was measured three times on each membrane to obtain the average density values of the polymers. The experiments were conducted at 20 °C.

### 2.10. Membrane Preparation

Composite polymer membranes with a selective layer based on synthesized PILs were obtained by casting corresponding solutions (2% in DMSO) onto a commercial microfiltration fluoropolymer membrane MFFK using an automatic casting knife MemcastTM Plus (POROMETER, Nazareth, Belgium) with a 100 μm blade followed by solvent evaporation in enclosed area under ambient temperature. After major solvent evaporation membranes were dried under vacuum to a constant weight.

### 2.11. Scanning Electron Microscopy (SEM)

Topography and morphological characteristics of the obtained composite membranes were studied by scanning electron microscopy (SEM) using an JSM-IT300LV electron microscope (JEOL, Peabody, MA, USA) with an electron probe diameter of about 5 nm and a probe current of less than 0.5 nA (operating voltage 20 kV). SEM scanning was performed using low-energy secondary electrons and backscattered electrons under a low vacuum to eliminate the charge. Supplementary Information for SEM images is listed in Table S3.

### 2.12. Wettability Measurements and Surface Free Energy Calculation

Wettability tests and surface energy calculation were performed according to a previously published technique [55]. The contact angle of wetting (θ) with three test liquids with different surface tensions (Table 2): water, glycerol, and diiodomethane was measured at an equilibrium state in a closed beaker and calculated using ImageJ software with a contact angle plugin based on experimental data. 

The measurements were conducted at 293 K. The results were collected for a series of five drops with contact angle deviations that did not exceed ± 1°. Total surface free energy and its components were calculated using the Owens-endt method. According to the Owens-Wendt method, the surface free energy $\left(\gamma_{s}\right)$ can be calculated as a sum of its dispersive ($\gamma_{s}^{d}$) and polar ($\gamma_{s}^{p}$) components:

(1) $$\gamma_{s}=\gamma_{s}^{d}+\gamma_{s}^{p}$$

These values can be graphically obtained from the Owens-Wendt equation using the results of surface wettability measurements with three different testing liquids obtained previously:

(2) $$\frac{\gamma_{l}\left(cos+1\right)}{2{\left(\gamma_{l}^{d}\right)}^{1/2}}={\left(\gamma_{s}^{p}\right)}^{1/2}\frac{{\left(\gamma_{l}^{p}\right)}^{1/2}}{{\left(\gamma_{l}^{d}\right)}^{1/2}}+{\left(\gamma_{s}^{d}\right)}^{1/2}$$

Here, $\gamma_{l}$ is the surface tension of the wetting liquid (mJ·m^−2^), $\gamma_{l}^{d}$ is a dispersive component of the surface tension of the wetting liquid (mJ·m^−2^), and $\gamma_{l}^{p}$ is a polar component of the surface tension of the wetting liquid (mJ·m^−2^).

### 2.13. Gas Permeation Tests

**Figure 1:**
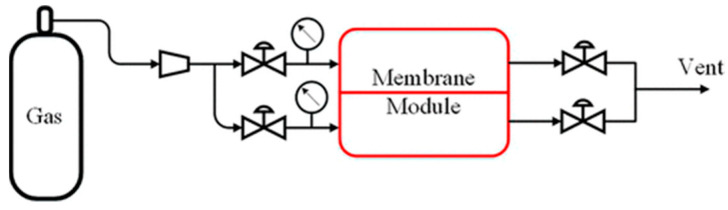
Experimental gas permeation setup with a membrane module.

For the gas permeation test, three individual gases, nitrogen (N_2_), carbon dioxide (CO_2_), and methane (CH_4_), were chosen to characterize the separation performance of the obtained membranes in natural gas and flue gas treatment. The presence of water influences the performance of ILs and related substances as well as the mechanism of CO_2_ solubility and transport [56,57,58,59]. In this regard, the presence of water in membranes was measured using infrared moisture determination balance FD-610 (Kett, Tokyo, Japan). The membrane sample (5.5 g) was placed in the balance and dried at 120 °C to a constant weight. The presence of water in all membranes was less than 0.1%. All gases used were 99.9% purity with content of water in CO_2_ at 0.001%; in N_2_, 0.007%; and in CH_4_, 0.0001%. The pure gas permeabilities of N_2_, CO_2_, and CH_4_ through the polymeric membranes were measured by an experimental setup (Figure 1) supported by an automatic computing system based on a software-logic controller (Unitronix, Israel) at the initial transmembrane pressure of 130 kPa and ambient temperature (293 K) in a constant volume mode [15].

Each single-gas test was repeated at least three times. The selected experimental curve of CO_2_ pressure time course in a permeation cell obtained for a membrane with a selective layer based on pVBmimTf_2_N is represented in Figure S7. Permeability coefficients were calculated according to the following formula [60]:

(3) $$\frac{1}{\beta }\ln\left(\frac{|p_{feed}-p_{perm}{|}_{0}}{\left|p_{feed}-p_{perm}\right|}\right)=\frac{1}{\beta }\ln\left(\frac{\Delta p_{0}}{\Delta p}\right)=P\frac{t}{l}$$

where *β* is a geometric setup parameter (m^−1^), $p_{feed}$ is the pressure in a high-pressure compartment (Pa), $p_{perm}$ is the pressure in a low-pressure compartment (Pa), *P* is the permeability coefficient (Barrer), *t* is time (s), and *l* is the selective layer thickness in a composite membrane (m). The ideal selectivity of the polymeric membranes was calculated as the ratio of the single gas permeability coefficients (gases A and B):

(4) $$\alpha_{A/B}=\frac{P_{A}}{P_{B}}$$

The ideal selectivity gives some idea of the membrane separation properties. However, this value is based on a simplified model of gas adsorption and diffusion in the mass-transfer process. In this regard, a true selectivity might differ when comes to a gas mixture.

## 3. Results and Discussion

### 3.1. PILs Identification and Properties

It was previously shown by L. C. Tomé [42] that PIL properties strongly depend on the polymer’s molecular weight. Medium-molecular-weight polymers (~200 kDa) demonstrated optimal thermal, mechanical, and transport properties. In the current case, PILs were synthesized by the functionalization of a polymer precursor—pVBCl. The results of pVBCl molecular weight and polydispersity determined by GPC are represented in Table 3.

pVBCl synthesized by radical polymerization is characterized by a high polydispersity expected for a radical process without the use of chain transfer agents. Since functionalization does not significantly influence the length of polymer chains, PILs’ molecular weight could be defined as medium (~200 kDa).

PILs synthesis includes stages of tertiary amine quaternization and anion exchange. Both reactions are complicated by a sterical hindrance related to the polymeric nature of the substrate. This leads to incomplete functionalization of the initial pVBCl. FD and ExD, determined conductometrically, are listed in Table 4.

A notable difference in ExD is observed for PILs containing Tf_2_N anions if compared with BF_4_ and PF_6_. For both pVBmimTf_2_N and pVBPyPyTf_2_N, ExD is much higher, which might be caused by the formation of a polymer with a loosened structure that facilitates an ion exchange reaction. 

**Figure 2:**
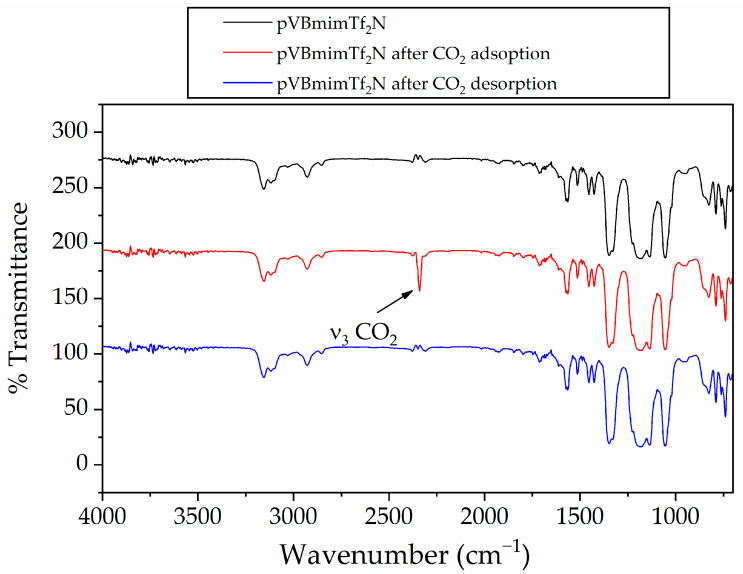
FTIR spectra of pure pVBmimTf_2_N, pVBmimTf_2_N after CO_2_ adsorption, and pVBmimTf_2_N after CO_2_ desorption.

**Figure 3:**
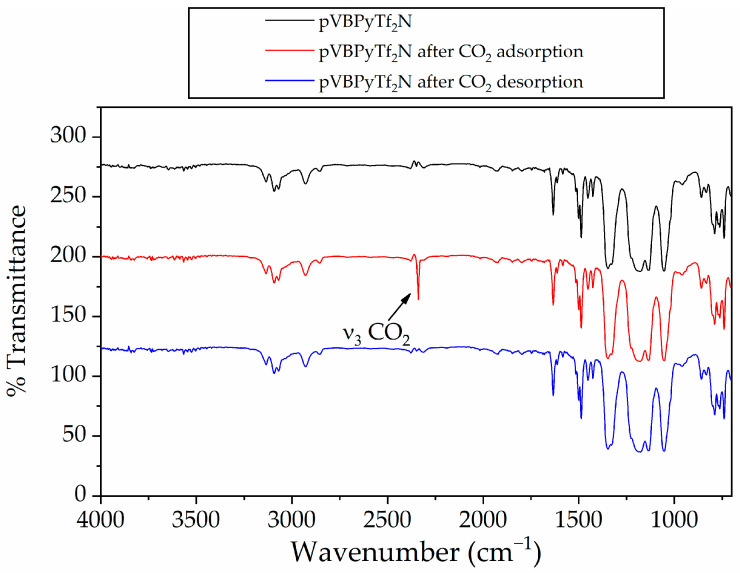
FTIR spectra of pure pVBPyTf_2_N, pVBPyTf_2_N after CO_2_ adsorption, and pVBPyTf_2_N after CO_2_ desorption.

The formation of PILs was confirmed by a combination of ^1^H NMR and ATR-FTIR spectroscopy. The data is represented in Supplementary Materials in Table S2 for ^1^H NMR and in Figures S1–S6 for ATR-FTIR. ATR-FTIR spectra for PILs containing Tf_2_N anion are represented in Figure 2 and Figure 3. After exposure to the CO_2_ atmosphere, a strong CO_2_ asymmetric stretch (ν_3_) bond is observed for PIL spectra in the region 2333–2341 cm^−1^.

Table 5 compares the position of the ν_3_ CO_2_ bond after interaction with PILs with those of free CO_2_ of solid and gas states. It is worth noting that ν_3_ CO_2_ in the PILs spectra (2339 cm^−1^) shifts for ~10 cm^−1^ in comparison to free carbon dioxide in a gas phase (2349 cm^−1^), which confirms the emergence of interaction between CO_2_ and PILs. A small value of shift is in favor of a physical nature of interaction and not chemical. The disappearance of the ν_3_ CO_2_ band caused by the quick desorption during vacuum treatment without a temperature increase is in the line with abovementioned observations. Thus, it could be concluded that the interaction between carbon dioxide and PILs is that of a physisorption type. However, ILs with Tf_2_N and carboxylate anion demonstrate chemosorption of CO_2_ [56,58]. The position of the bond varies depending on the nature of the anion in polyelectrolyte. The frequency of the ν_3_ CO_2_ band demonstrates the most significant shift (14 cm^−1^) when PILs with Cl-anion are considered. Apparently, it proceeds due to the higher polarizing effect of small Cl-anion in comparison to other investigated anions with a larger radius. In the case of gas separation membranes, weaker interaction is preferable because it provides faster sorption–desorption acts in a mass-transfer process acting by the solution–diffusion mechanism.

Further, the density of polymer films obtained in the same conditions as a selective layer of composite membranes on a glass support was evaluated. 

**Figure 4:**
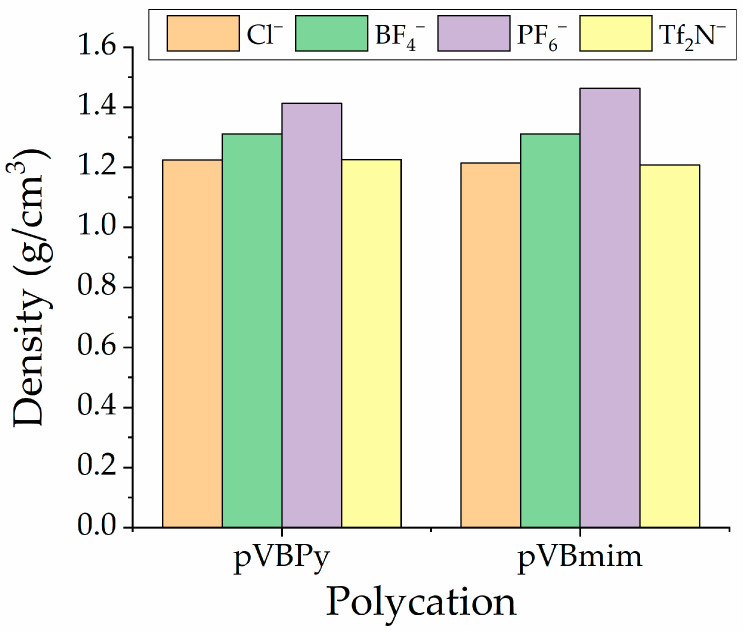
Density of nonporous polymer films based on synthesized PILs.

As seen in Figure 4 and in Table S1, the density of the nonporous films based on the synthesized PILs are higher than those for initial pVBCl (1.1889 g/cm^3^) in all cases. The increase of the PILs’ density compared to that of pVBCl can be addressed to the close package of macromolecular chains in the polymer matrix due to intermolecular electrostatic interactions. It was observed that density is insignificantly influenced by the type of polycation and, to a greater extent, depends on a type of counterion. The increasing of PILs’ density values follows the consequence of PIL anion Cl^−^~Tf_2_N^−^ < BF_4_^−^ < PF_6_^−^. Based on this correlation, it was supposed that the free volume in PILs has a likewise dependence. A relatively low density of polyelectrolytes with Tf_2_N^−^ anion supports the assumption that ion exchange reaction in this case would result in a higher yield. Since gas separation processes in the case of nonporous polymeric membranes follow the solution–diffusion mechanism and the process is limited by a diffusion of penetrants in the polymer matrix, lower-density polymers are expected to be more permeable.

### 3.2. Membrane Characterization 

**Figure 5:**
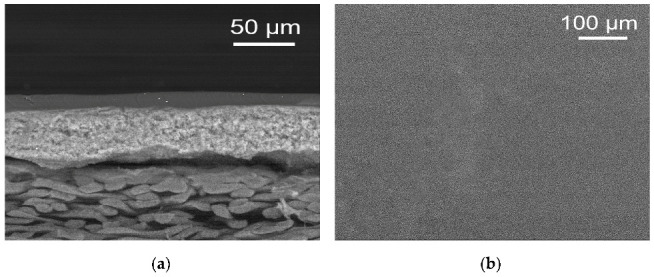
SEM microphotographs of pVBCl/MFFK cross-section (**a**) and surface (**b**).

**Figure 6:**
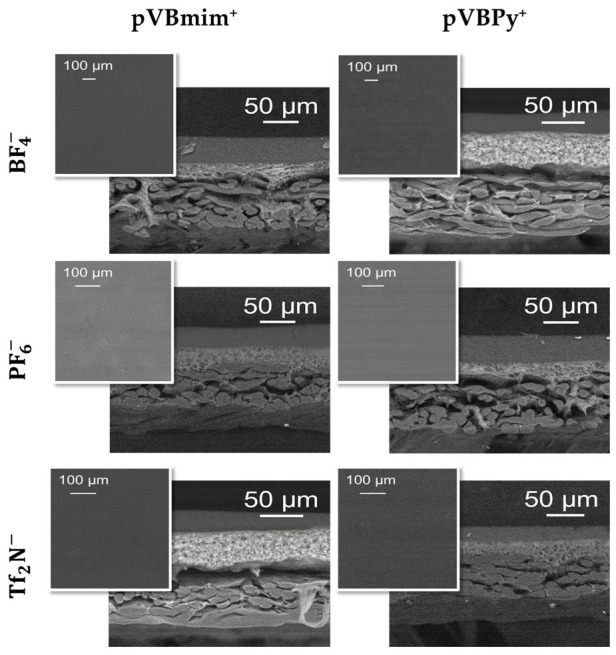
SEM microphotographs of PILs/MFFK cross-section and surface.

Most previously reported PILs are characterized by low breaking strength and high fragility [28,42,46,51]. This drawback could be solved by developing a composite membrane with a porous support providing the required mechanical strength. Thereby, composite membranes composed of porous fluoropolymer support and a thin selective layer based on a synthesized polymers were developed. Membrane cross-sectional microstructure and surface topography registered by SEM are shown in Figure 5 and Figure 6.

According to SEM, three well-defined layers are observed on a cross-sectional SEM microphotograph from top to bottom: (i) the PIL selective layer, (ii) the porous fluoroplastic microfiltration layer, and (iii) the nonwoven fabric. Layers (ii) and (iii) belong to the commercial microfiltration membrane used as a support. PILs demonstrate good adhesion to the fluoroplastic layer. Penetration of PILs into the porous of the support is not observed. The values of selective layer thickness obtained from SEM cross-sectional microphotographs were used to calculate permeability coefficients for each PIL. The average thickness is about 50 micrometers, which is several times higher than in advanced reverse osmosis or pervaporation membranes. In this case, thick selective layer prevents mechanical defects that drastically influence membranes performance. Another way to provide mechanically stability to a thin PIL layer is mixing with compatible IL [63]. The obtained PILs form a homogeneous defect-free layer on the surface of porous fluoroplastic support. Comparison of SEM microphotographs of the surface for the initial pVBCl (Figure 5) with PILs (Figure 6) shows conformity. The flexible pVBCl backbone structure determines the appearance of the PIL surface in this case. Densely packed polymeric chains form uniform amorphous structures. Despite the PILs’ selective layers having a similar topography, the nature of ionic substitute in a polymer significantly influences the energetic characteristics of the membrane surface.

**Figure 7:**
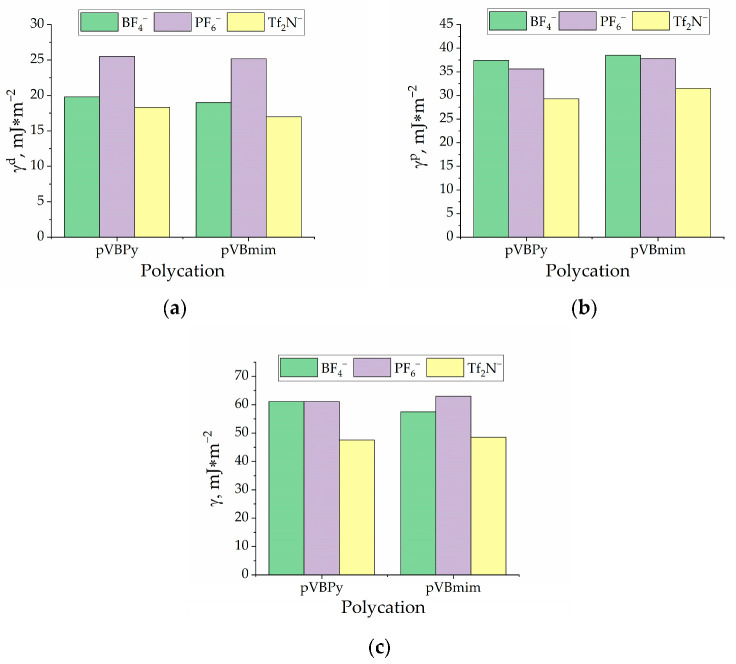
Surface free energy (dispersive (**a**), polar (**b**), and overall (**c**)) of the polymeric membranes.

The surface free energy and its polar and dispersive components were evaluated using the Owens–Wendt method based on wettability measurements. The results are shown in Figure 7.

PILs obtained by the functionalization of pVBCl combine properties of a hydrophobic polystyrene backbone with the hydrophilic properties of charged ionic moieties. Due to the amphiphilic nature of PILs, the surfaces of composite membranes are well wetted by the nonpolar test liquid diiodomethane as well as by water and glycerol; however, there are some differences in wettability as a result of different surface free energy, depending on the nature of the tertiary amine and counter ion. As was shown by Bogdanova and Dolzhikov [64] for glassy polymers the dispersive component is linearly correlated with the free volume of the polymer since both values are impacted by the density of pacing in the polymer matrix. The results of dispersive energy component calculation support the previously discussed results of the density measurements. The dispersive energy changes the same way as the density of PILs and follow the order: Tf_2_N^−^ < BF_4_^−^ < PF_6_^−^. The polar component of surface free energy is slightly lower for polycations with Tf_2_N^−^ counter ion in comparison to other PILs. Overall, the results demonstrate that polycationic PIL surface free energy is insignificantly altered by the type of tertiary amine in the polymer structure and, to a greater extent, depends on the nature of the anion.

**Figure 8:**
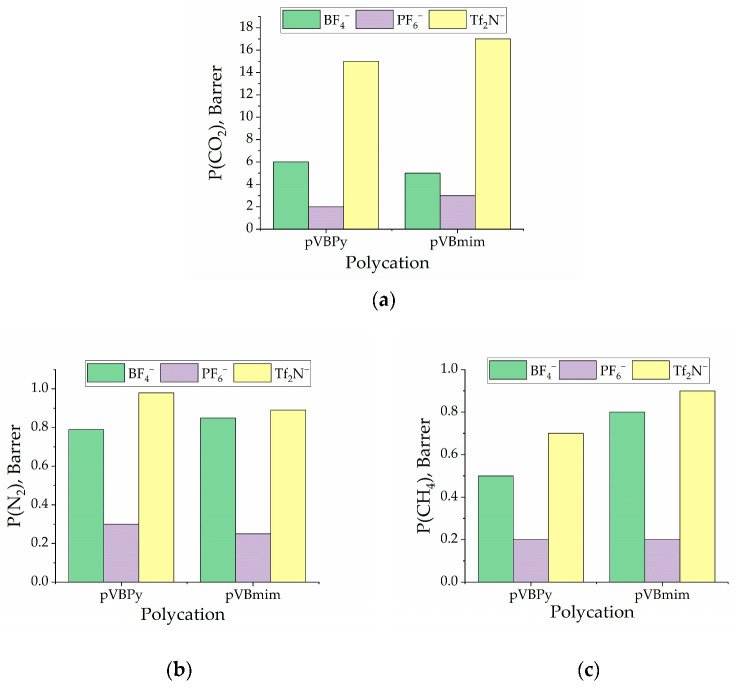
Permeability coefficients for the polymeric membranes based on PILs: (**a**) for CO_2_, (**b**) for N_2_, and (**c**) for CH_4_.

The density of the polymer and its surface properties are in a close correlation with gas transport properties. According to the results of density measurements and surface free energy calculations, a greater permeability of Tf_2_N^−^-containing PILs was expected and confirmed experimentally. The permeability coefficients of the obtained membranes are represented in Figure 8.

**Figure 9:**
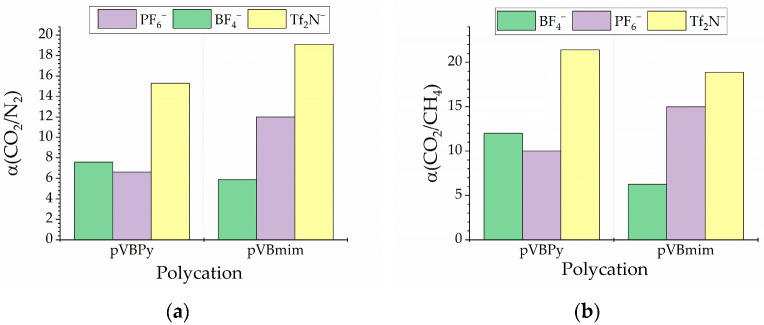
Ideal selectivity for pair of gases. (**a**) CO_2_/N_2_, (**b**) CO_2_/CH_4_.

The data on the density and energy of the surface is consistent with the results of the gas separation. pVBPyTf_2_N and pVBmimTf_2_N have a lower density and, as a consequence, a larger free volume and lower surface energy, which turned out to be more permeable to all gases. While PILs containing PF_6_ anions are the least permeable, which agrees with the density measurements. Naturally, polymers with a high density (pVBPyPF_6_ and pVBmimPF_6_) have improved selectivity. Figure 9 shows the comparison of ideal selectivity for composite membranes composed of PILs.

PILs containing Tf_2_N^−^ anions display an optimal combination of permeability and selectivity in comparison to other anions. 

## 4. Conclusions

PILs have recently emerged as a promising platform for various separation applications due to a combination of their polymeric nature with a unique property of ILs—the ability to capture CO_2_. In this study, the design and performance of composite membranes based on imidazolium and pyridinium polycationic salts with different anions, such as hexafluorophosphate, tetrafluoroborate, and bis(trifluoromethylsulfonyl)imide, were investigated. PILs were synthesized using a simple ion exchange reaction from a basic polymer precursor, pVBCl. It was found that the functionalization degree and anion exchange degree depend on the nature of substitute. The formation of PILs was confirmed by a combination of NMR and ATR-FTIR spectroscopy. ATR-FTIR was also used to characterize PILs interaction with CO_2_. Several studies [56,58] have previously reported chemosorption of CO_2_ by ILs containing Tf_2_N and carboxylate anions. Spectroscopic data on PILs interaction with CO_2_ is more in favor of physisorption in the case of polymerized ionic liquids. Properties of the synthetized PILs and gas separation properties of PIL-based composite membranes are summarized in Table 6.

In general, PILs showed properties comparable to the previously reported (Table 1) properties and could be further investigated as a membrane matrix material. PILs that contain Tf_2_N^−^ anions exhibited superior separation performance compared to other polymers. The further investigation of pVBCl-based gas separation membranes could be focused on, combining polycations with carboxylate anions that also show a good CO_2_ absorption [56,58], as well as the influence of water presence on the membrane performance. 

## Figures and Tables

**Table 1:** Gas separation properties of membranes synthesized of VBCl homopolymer derivatives.

PIL Formula	P ^a^ (CO_2_), Barrer ^d^	P (N_2_), Barrer	P (CH_4_), Barrer	A ^b^ (CO_2_/N_2_)	α (CO_2_/CH_4_)	References
p[VBimOEG_1_ *][Tf_2_N]	16 ± 1	0.39 ± 0.02	0.48 ± 0.01	41	33	[46]
P[VBimOEG_2_ *][Tf_2_N]	22 ± 1	0.50 ± 0.01	0.74 ± 0.02	44	29	
p[VBimC_3_CN][Tf_2_N]	4.1 ± 0.1	0.11 ± 0.01	0.11 ± 0.01	37	37	
p[VBimC_5_CN][Tf_2_N]	8.2 ± 0.3	0.21 ± 0.01	0.28 ± 0.02	40	30	
p[VB(CH_2_CH_2_O)_2_CH_3_im][Tf_2_N]	22 ± 1	_	_	44	29	
p[VBmim][Tf_2_N]	9.2 ± 0.5	0.29 ± 0.01	0.24 ± 0.01	32	39	[27]
p[VBbim][Tf_2_N]	20 ± 1	0.67 ± 0.02	0.91 ± 0.06	30	22	
p[VBC_6_im][Tf_2_N]	32 ± 1	1.4 ± 0.1	2.3 ± 0.1	28	17	
**PIL Formula**	$\Pi_{CO_{2}}$ **^c^, GPU ^e^**	$\Pi_{N_{2}}$ **, GPU ^b^**		α ** (CO_2_/N_2_)		
p[VBTMA][Tf_2_N]	132.0 ± 44.0 GPU	5.0 ± 2.0 GPU	_	27.0 ± 1.3	_	[51]
p[VBHEDMA][Tf_2_N]	109.0 ± 0.5 GPU	2.6 ± 0.5 GPU	_	41.6 ± 0.6	_	
p[VBMP][Tf_2_N]	1334.0 ± 263.8 GPU	78.0 ± 15.5 GPU	_	17.2 ± 0.1	_	

^a^ P—individual gas permeability coefficient; ^b^ α—ideal selectivity; ^c^ Π—permeance; ^d^ Barrer = 10^−10^ cm^3^(STP)cmcm^−2^s^−1^cmHg^−1^; ^e^ GPU = 10^−6^ cm^3^(STP)cm^−2^s^−1^cmHg^−1^; * OEG—oligo(ethylene glycol); ** α—mixed gas selectivity.

**Table 2:** Surface tension values (overall, dispersive, and polar) of the test liquids.

Test Liquid	$\gamma_{l}^{d},\;mJ\cdot m^{-2}$	$\gamma_{l}^{p},\;mJ\cdot m^{-2}$	$\gamma_{l},\;mJ\cdot m^{-2}$
Water	19.9	52.2	72.1
Glycerol	37.0	26.4	63.4
Diiodomethane	49.5	1.3	50.8

**Table 3:** pVBCl molecular weight and polydispersity.

Polymer	Molecular Weight		Polydispersity
	M_n_, kDa	M_w_, kDa	
pVBCl	131	228	2.5

**Table 4:** Functionalization degree and anion exchange degree.

Polymer	FD, %	ExD, %	The Amount of IL Monomer Units per 1 g of PIL, mol/g
pVBPyCl	91	-	0.0045
pVBPyBF_4_	-	86.5	0.0030
pVBPyPF_6_	-	88.5	0.0023
pVBPyTf_2_N	-	97	0.0025
pVBmimCl	85	-	0.0041
pVBmimBF_4_	-	67.5	0.0022
pVBmimPF_6_	-	67	0.0016
pVBmimTf_2_N	-	90.5	0.0021

**Table 5:** The position of asymmetric CO_2_ stretching fundamental in PILs spectra.

Polymer	ν_3_ CO_2_, cm^−1^
pVBmimCl	2335
pVBmimBF_4_	2339
pVBmimPF_6_	2341
pVBmimTf_2_N	2339
pVBPyCl	2333
pVBPyBF_4_	2339
pVBPyPF_6_	2339
pVBPyTf_2_N	2339
**Reference**	**ν_3_ CO_2_, cm^−1^**
Gas [61]	2349
Crystalline solid, 77 K [62]	2344

**Table 6:** Properties of PILs and PILs-based membranes.

PIL Name	Density, g/cm^3^	Surface Free Energy, $mJ*m^{-2}$			P(N_2_), Barrer	P(CO_2_), Barrer	P(CH_4_), Barrer	α (CO_2_/N_2_)	α (CO_2_/CH_4_)
		$\gamma^{d}$	$\gamma^{p}$	γ					
pVBmimCl	1.2140	- *	-	-	-	-	-	-	-
pVBmimBF_4_	1.3115	19	38.5	57.5	0.8	5	0.8	5.88	6.25
pVBmimPF_6_	1.4635	25.2	37.8	62.9	0.2	3	0.2	12	15
pVBmimTf_2_N	1.2076	17	31.5	48.5	0.9	17	0.9	19.1	18.88
pVBPyCl	1.2240	-	-	-	-	-	-	-	-
pVBPyBF_4_	1.3111	19.8	37.4	57.5	0.8	6	0.5	7.6	12
pVBPyPF_6_	1.4132	25.5	35.6	62.9	0.3	2	0.2	6.6	10
pVBPyTf_2_N	1.2258	18.3	29.3	48.5	1	15	0.7	15.3	21.43

* “-”—was not measured.

## Data Availability

The authors confirm that the data supporting the findings of this study are available within the article and its Supplementary Materials.

## Supplementary Materials

The following supporting information can be downloaded at: https://www.mdpi.com/article/10.3390/membranes13060539/s1, Figure S1: ATR-FTIR spectra for pVBmimCl, Figure S2: ATR-FTIR spectra for pVBmimBF_4_, Figure S3: ATR-FTIR spectra for pVBmimPF_6_, Figure S4: ATR-FTIR spectra for pVBPyCl, Figure S5: ATR-FTIR spectra for pVBPyBF_4_, Figure S6: ATR-FTIR spectra for pVBPyPF_6_, Figure S7: The time course of CO_2_ pressure in permeation cell for a membrane with pVBmimTf_2_N selective layer; Table S1: Mixtures of liquids used for PILs density evaluation, Table S2: NMR data, Table S3: Supplementary Information for SEM images
