# Supplementary material for: The Influence of Polycation and Counter-Anion Nature on the Properties of Poly(ionic liquid)-Based Membranes for CO2 Separation
Source: Membranes (Basel). 2023 May 23;13(6):539. doi: 10.3390/membranes13060539 (PMC10304824; doi:10.3390/membranes13060539)
Supplement: Supplementary file 1 [file membranes-13-00539-s001.zip › membranes-2361950-supplementary.pdf]

Supplementary materials for

The Influence of Polycation and Counter-anion Nature on the Properties of Poly(ionic liquid)-Based Membranes for CO_2 Separation

Ksenia V. Otvagina [1], Alexey A. Maslov [1], Diana G. Fukina [2], Anton N. Petukhov [1,3], Yulia B. Malysheva [4], Andrey V. Vorotyntsev [1], Tatyana S. Sazanova [3,5,6], Artem A. Atlaskin [3], Alexander A. Kapinos [1], Alexandra V. Barysheva [1], Sergey S. Suvorov [1], Ivan D. Zanozin [1], Egor S. Dokin [1], Ilya V. Vorotyntsev [3] and Olga V. Kazarina [1,5,6,*]

1 Chemical Engineering Laboratory, Research Institute for Chemistry, N.I. Lobachevsky State University of Nizhny Novgorod, 23 Gagarin Avenue, 603950 Nizhny Novgorod, Russia; k.v.otvagina@gmail.com (K.V.O.); antopetukhov@gmail.com (A.N.P.); an.vorotyntsev@gmail.com (A.V.V.); kapinos98@gmail.com (A.A.K.); aleksandra.barysheva@gmail.com (A.V.B.); e-dokin@yandex.ru (E.S.D.)
2 Research Institute for Chemistry, N.I. Lobachevsky State University of Nizhny Novgorod, 23 Gagarin Avenue, 603950 Nizhny Novgorod, Russia; dianafuk@yandex.ru
3 Laboratory of SMART Polymeric Materials and Technologies, Mendeleev University of Chemical
Technology, 9 Miusskaya Square, 125047 Moscow, Russia; atlaskin@gmail.com (A.A.A.); ilyavorotyntsevv@gmail.com (I.V.V.)
4 Organic Chemistry Department, N.I. Lobachevsky State University of Nizhny Novgorod, 23 Gagarin Avenue, 603950 Nizhny Novgorod, Russia
5 Laboratory of Membrane and Catalytic Processes, Nizhny Novgorod State Technical University n.a. R.E. Alekseev, 24 Minin Street, 603950 Nizhny Novgorod, Russia
6 Laboratory of Ionic Materials, Mendeleev University of Chemical Technology, 9 Miusskaya Square, 125047 Moscow, Russia
* Correspondence: olga_kazarina@list.ru; Tel.: +7-920-001-7305

To evaluate the densities of PILs, mixtures of liquids with different densities were used. In Table S1 components of the mixtures are listed for each PIL.

Table S1. Mixtures of liquids used for PILs density evaluation.

Polymer	Component 1	ϱ@20°C, g/cm³	Component 2	ϱ@20°C, g/cm³
pVBCl	water	0.9982	glycerol	1.2636
pVBPyCl	dioxane	1.0363	chloroform	1.4892
pVBmimCl	dioxane	1.0363	chloroform	1.4892
$pVBPyBF_4$	dioxane	1.0363	chloroform	1.4892
$pVBmimBF_4$	dioxane	1.0363	chloroform	1.4892
$pVBPyPF_6$	chloroform	1.4892	carbon tetrachloride	1.5942
$pVBmimPF_6$	chloroform	1.4892	carbon tetrachloride	1.5942
$pVBPyTf_2N$	dioxane	1.0363	chloroform	1.4892
$pVBmimTf_2N$	dioxane	1.0363	chloroform	1.4892

All synthesized PILs were identified by means of Nuclear Magnetic Resonance Spectroscopy (NMR) and Attenuated Total Reflectance Fourier Transforms Infrared Spectroscopy

(ATR-FTIR). NMR data is represented in Table S1 and ATR-FTIR spectra are shown in Figures S1-S6.

Table S2. NMR data

**pVBmimCl** ^{1}H NMR (400 MHz, D_2O, δ in ppm): 8.89 (br s, 1H, ArH imidazole), 7.47 – 7.01 (br m, 2H, ArH imidazole + 2H, ArH), 6.58 (br s, 2H, ArH), 5.33 (br s, 2H, C_6H_4-C*H*$_2$-N), 3.79 (br s, 3H, CH_3), 2.05 – 0.90 (br m, 3H, –CH_2–CH–). FT-IR (KBr), n (cm^{-1}): 3145, 3077, 3027, 2923,2852 (CH_2, CH), 1572, 1559, 1512, 1453, 1423, 1384 (C = C, C = N). M$_n$=27300; PDI=1.86.
**pVBmimBF$_4$** ^{1}H NMR (400 MHz, DMSO-d$_6$, δ in ppm): δ 9.04 (br s, 1H, ArH imidazole), 7.75 – 7.22 (br m, 2H, ArH imidazole), 7.07 (br s, 2H, ArH), 6.43 (br s, 2H, ArH), 5.22 (br s, 2H, C_6H_4-C*H*$_2$-N), 3.81 (br s, 3H, CH_3), 2.35 – 0.31 (m, 3H, –CH_2–CH–). FT-IR (KBr), n (cm^{-1}): 3159, 3115, 3028, 2926, 2852 (CH_2, CH), 1573, 1515,1457, 1427, 1389 (C = C, C = N), 1050 (BF_4). M_n = 33200; PDI = 2.04.
**pVBmimPF$_6$** ^{1}H NMR (400 MHz, DMSO-d$_6$, δ in ppm): δ 9.75 (br s, 1H, ArH imidazole), 7.59 – 7.97 (br m, 2H, ArH imidazole), 7.25 (br s, 2H, ArH), 6.34 (br s, 2H, ArH), 5.46 (s, 2H, C_6H_4-C*H*$_2$-N), 3.85 (s, 3H, CH_3), 1.93 – 0.78 (br m, 3H, –CH_2–CH–). FT-IR (KBr), n (cm^{-1}): 3162, 3119, 3031, 2927, 2853 (CH_2, CH), 1576, 1563, 1515, 1455, 1427, 1392 (C = C, C = N), 830, 560 (PF_6). M_n = 39900; PDI = 2.04.
**pVBmimTf$_2$N** ^{1}H NMR (400 MHz, DMSO-d$_6$, δ in ppm): δ 9.19 (br s, 1H, ArH imidazole), 7.62 (br s, 1H, ArH imidazole), 7.45 (br s, 1H, ArH imidazole), 7.00 (br s, 2H, ArH), 6.38 (s, 2H, ArH), 5.29 (br s, 2H, C_6H_4-C*H*$_2$-N), 3.83 (br s, 3H, CH_3), 1.84 – 0.85 (br m, 3H, –CH_2–CH–). FT-IR (KBr), n (cm^{-1}): 3163, 3118, 3097, 3031, 2961, 2929, 2852 (CH_2, CH), 1577, 1561,1516,1457, 1429 (C = C, C = N), 1354, 1196, 1137, 1058 (Tf_2N). M_n = 55700; PDI = 2.04.
**pVBPyCl** ^{1}H NMR (400 MHz, D_2O, δ in ppm). 8.89 (br s, 2H, *o*-H, C_6H_5N), 8.44 – 8.18 (br m, 1H, *p*-H, C_6H_5N), 8.01 – 7.72 (m, 2H, *m*-H, C_6H_5N), 7.27 (s, 2H, ArH), 6.53 (s, 2H, ArH), 5.78 (s, 2H, C_6H_4-C*H*$_2$-N), 1.46 (br m, 3H, –CH_2–CH–). FT-IR (KBr), n (cm^{-1}): 3428 (H_2O), 3128, 3088, 3065, 2924, 2855 (CH_2, CH), 1612, 1580, 1501, 1485, 1450, 1426 (C = C). M$_n$ = 20200; PDI=1.86.
**pVBPyBF$_4$** ^{1}H NMR (400 MHz, DMSO-d$_6$, δ in ppm): 9.09 (br s, 2H, *o*-H, C_6H_5N), 8.54 (br s, 1H, *p*-H, C_6H_5N), 8.10 (br s, 2H, *m*-H, C_6H_5N), 7.21 (br s, 2H, ArH), 6.45 (d br s, 2H, ArH), 5.74 (br s, 2H, C_6H_4-C*H*$_2$-N), 1.78 – 0.77 (m, 3H –CH_2–CH–). FT-IR (KBr), n (cm^{-1}):

	3428 (H_2O), 3138, 3100, 3053, 2929, 2854 (CH_2, CH), 1614, 1582, 1505, 1489, 1452, 1426 (C = C), 1054 (BF_4). M_n = 24700; PDI = 1.86.
	**pVBPyPF$_6$** ^{1}H NMR (400 MHz, DMSO-d$_6$, δ in ppm): 9.35 – 8.97 (m, 2H, *o*-H, C_6H_5N), 8.64 – 8.42 (m, 1H, *p*-H, C_6H_5N), 8.23 – 7.98 (m, 2H, *m*-H, C_6H_5N), 7.21 (br s, 2H, ArH), 6.35 (br s, 2H, ArH), 5.77 (br s, 2H, C_6H_4-C*H*$_2$-N), 1.76 – 0.73 (m, 3H –CH_2–CH–). FT-IR (KBr), n (cm^{-1}): 3428 (H_2O), 3141, 3100, 3070, 2928, 2854 (CH_2, CH), 1614, 1585, 1503, 1490, 1453, 1429 (C = C), 828, 557 (PF_6). M_n = 29800; PDI = 1.86.
	**pVBPyTf$_2$N** ^{1}H NMR (400 MHz, DMSO-d$_6$, δ in ppm): 9.00 (br s, 2H, *o*-H, C_6H_5N), 8.57 (br s, 1H, *p*-H, C_6H_5N), 8.11 (br s, 2H, *m*-H, C_6H_5N), 7.07 (br s, 2H, ArH), 6.32 (br s, 2H, ArH), 5.64 (br s, 2H, C_6H_4-C*H*$_2$-N), 2.16 – 0.44 (m, 3H –CH_2–CH–). FT-IR (KBr), n (cm^{-1}): 3428 (H_2O), 3141, 3095, 3053, 2927, 2855 (CH_2, CH), 1612, 1582, 1501, 1488, 1455, 1431 (C = C), 1361, 1194, 1136, 1058 (Tf_2N). M_n =41600; PDI=1.86.

ATR-FTIR spectra represents the results obtained for pure PILs as well as for PILs after exposure to CO_2 and after CO_2 desorption. Spectra for pVBmimTf$_2$N and pVBPyTf$_2$N are shown in the main text.

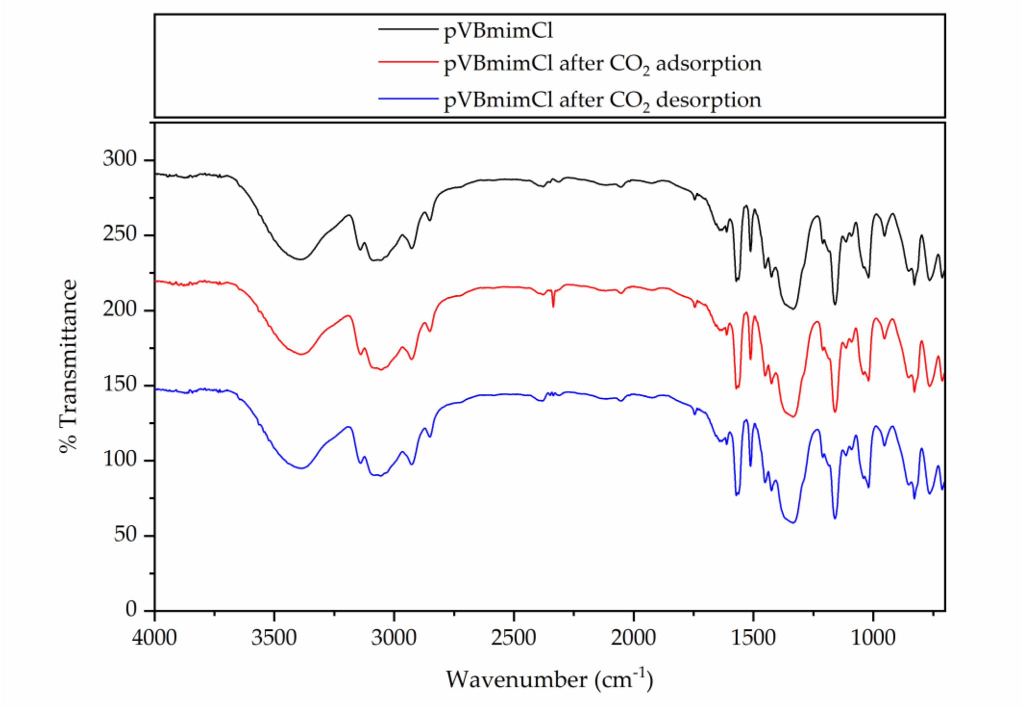

Figure S1. ATR-FTIR spectra for pVBmimCl

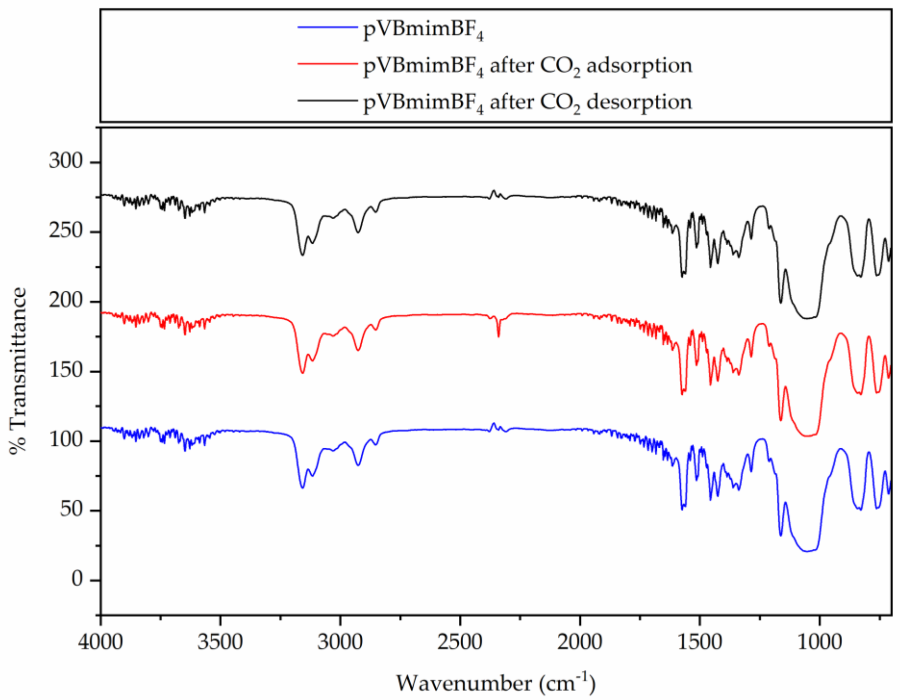

Figure S2. ATR-FTIR spectra for pVBmimBF$_4$

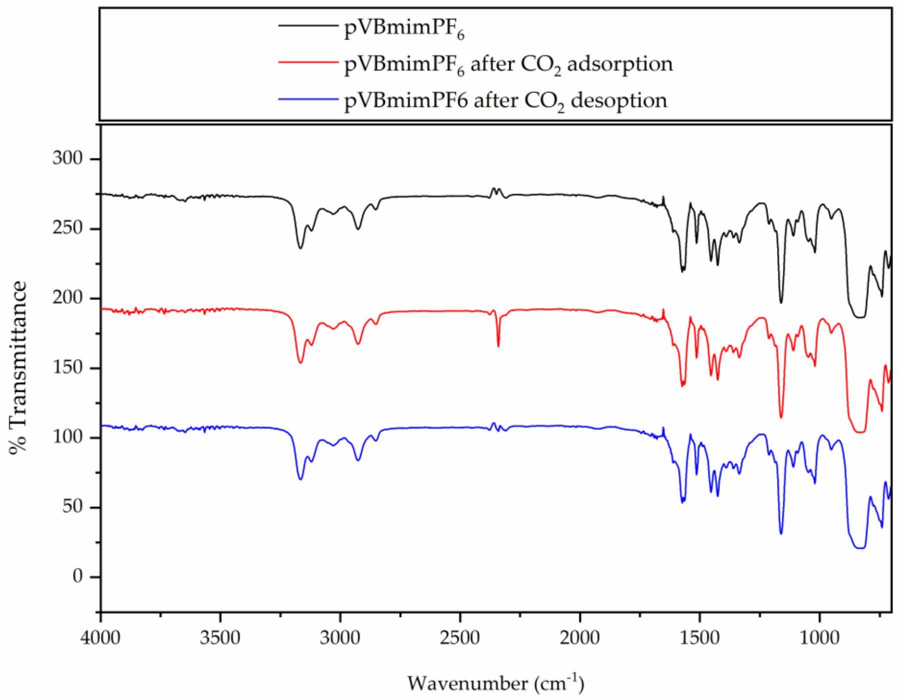

Figure S3. ATR-FTIR spectra for pVBmimPF$_6$

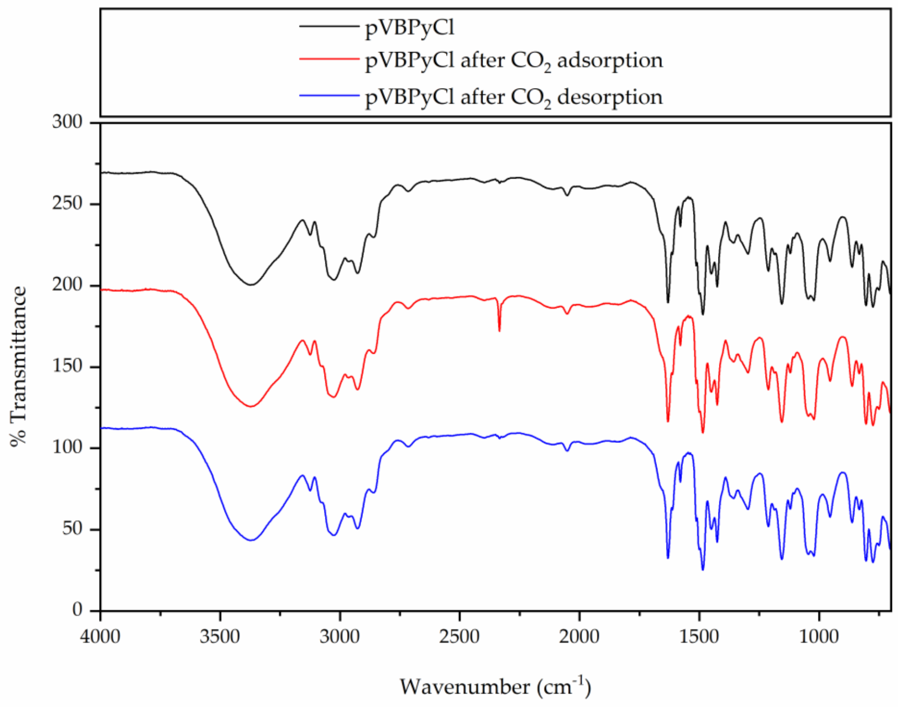

Figure S4. ATR-FTIR spectra for pVBPyCl

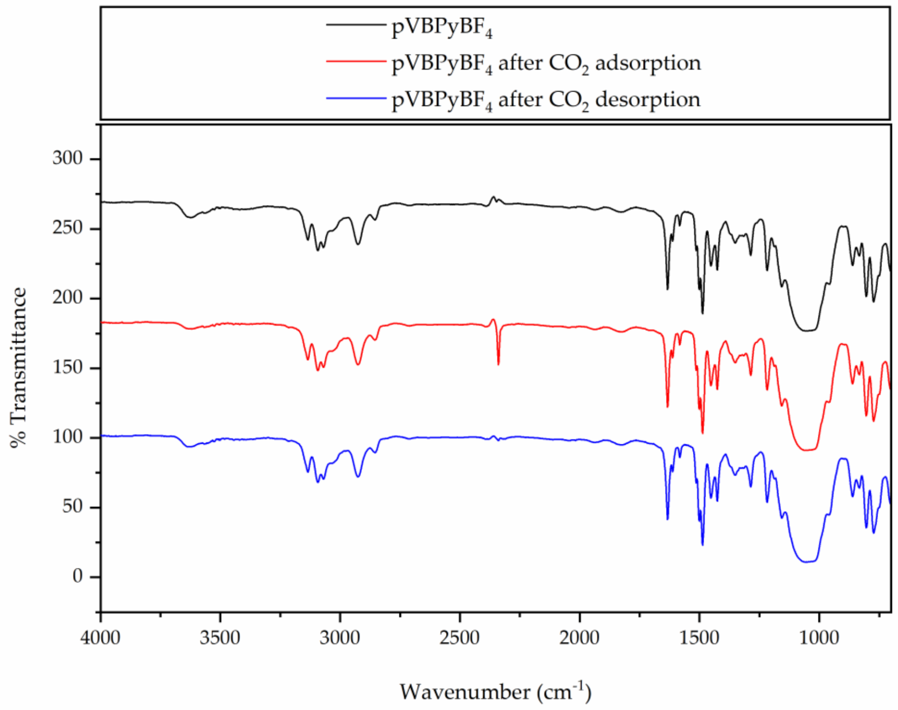

Figure S5. ATR-FTIR spectra for $pVBPyBF_4$

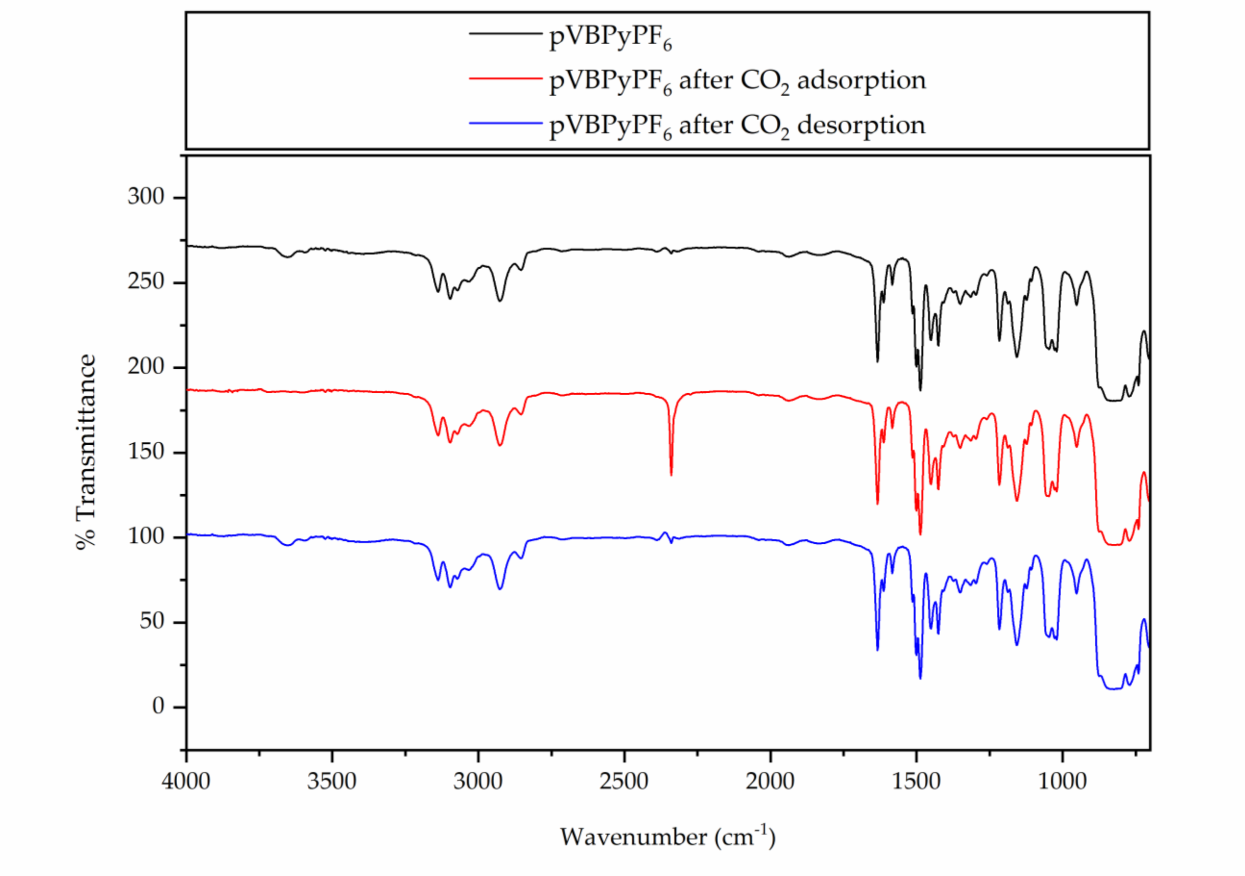

Figure S6. ATR-FTIR spectra for $pVBPyPF_6$

The selected experimental data of time course of pressure difference in permeation cell is represented in Figure S7.

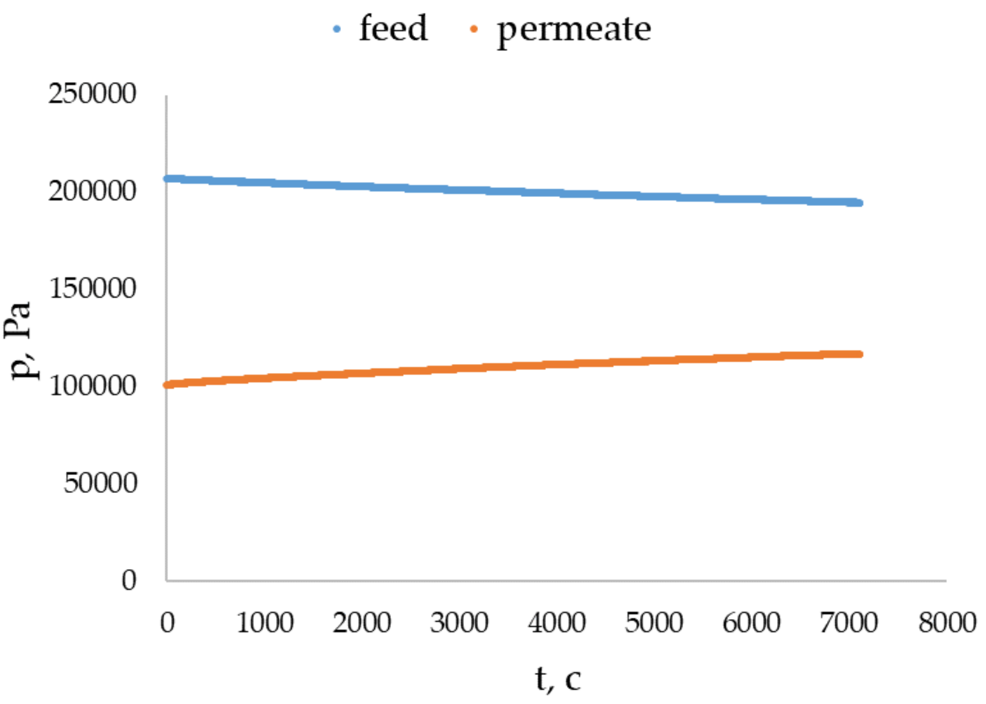

Figure S7. The time course of CO_2 pressure in permeation cell for a membrane with $pVBmimTf_2N$ selective layer.

Supplementary information for SEM images is listed in Table S3.

Table S3. Supplementary information for SEM images.

Polymer	Information for cross-section image	Information for surface image
pVBC	BET-C, operating voltage 20 kV, WD 14.80 mm x200, High-P.C.15.0, High vac.	BET-C, operating voltage 20 kV, WD 10.00 mm x200, High-P.C.15.0, High vac.
pVBmimBF$_4$	BET-C, operating voltage 20 kV, WD 11.30 mm x200, High-P.C.30.0, 40 Pa.	BET-C, operating voltage 20 kV, WD 9.7 mm x100, High-P.C.30.0, 39 Pa.
pVBmimPF$_6$	BET-C, operating voltage 20 kV, WD 10.10 mm x200, High-P.C.30.0, 41 Pa.	BET-C, operating voltage 20 kV, WD 9.8 mm x200, High-P.C.30.0, 40 Pa.
pVBmimTf$_2$N	BET-C, operating voltage 20 kV, WD 10.40 mm x200, High-P.C.30.0, 40 Pa.	BET-C, operating voltage 20 kV, WD 9.9 mm x200, High-P.C.15.0, High vac.
pVBPyBF$_4$	BET-C, operating voltage 20 kV, WD 8.80 mm x200, High-P.C.20.0, 45 Pa.	BET-C, operating voltage 20 kV, WD 9.8 mm x100, High-P.C.40.0, 45 Pa.
pVBPyPF$_6$	BET-C, operating voltage 20 kV, WD 8.50 mm x200, High-P.C.20.0, 45 Pa.	BET-C, operating voltage 20 kV, WD 9.9 mm x200, High-P.C.30.0, 44 Pa.
pVBPyTf$_2$N	BET-C, operating voltage 20 kV, WD 8.80 mm x200, High-P.C.20.0, 45 Pa.	BET-C, operating voltage 20 kV, WD 9.8 mm x200, High-P.C.40.0, 44 Pa.
